# Supplementary figures and images for: Improved plaque assay for human coronaviruses 229E and OC43
Source: PeerJ. 2020 Dec 21;8:e10639. doi: 10.7717/peerj.10639 (PMC7759117; doi:10.7717/peerj.10639)

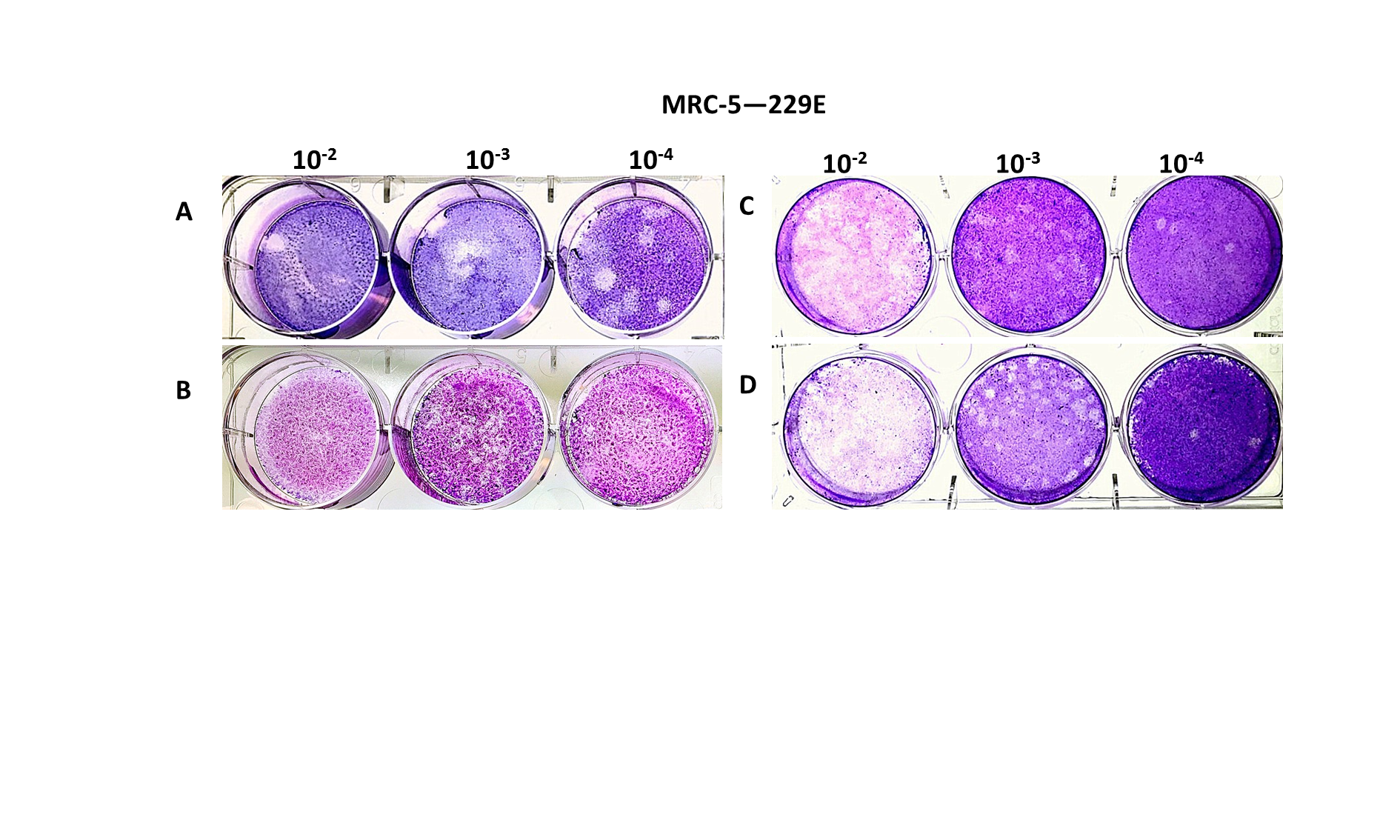

Supplement: Supplemental Information 1 — Plaques formed at 5 dpi for 229E on MRC-5 monolayer with inoculum left and the EMEM-based overlay mediums with following indicated final concentrations: (A) 0.3% of agarose and 5% of FBS; (B) 0.5% of agarose and 8% of FBS; (C) 0.75% of agarose and 8% of FBS; (D) 0.75% of agarose and 5% of FBS. [file peerj-08-10639-s001.png]

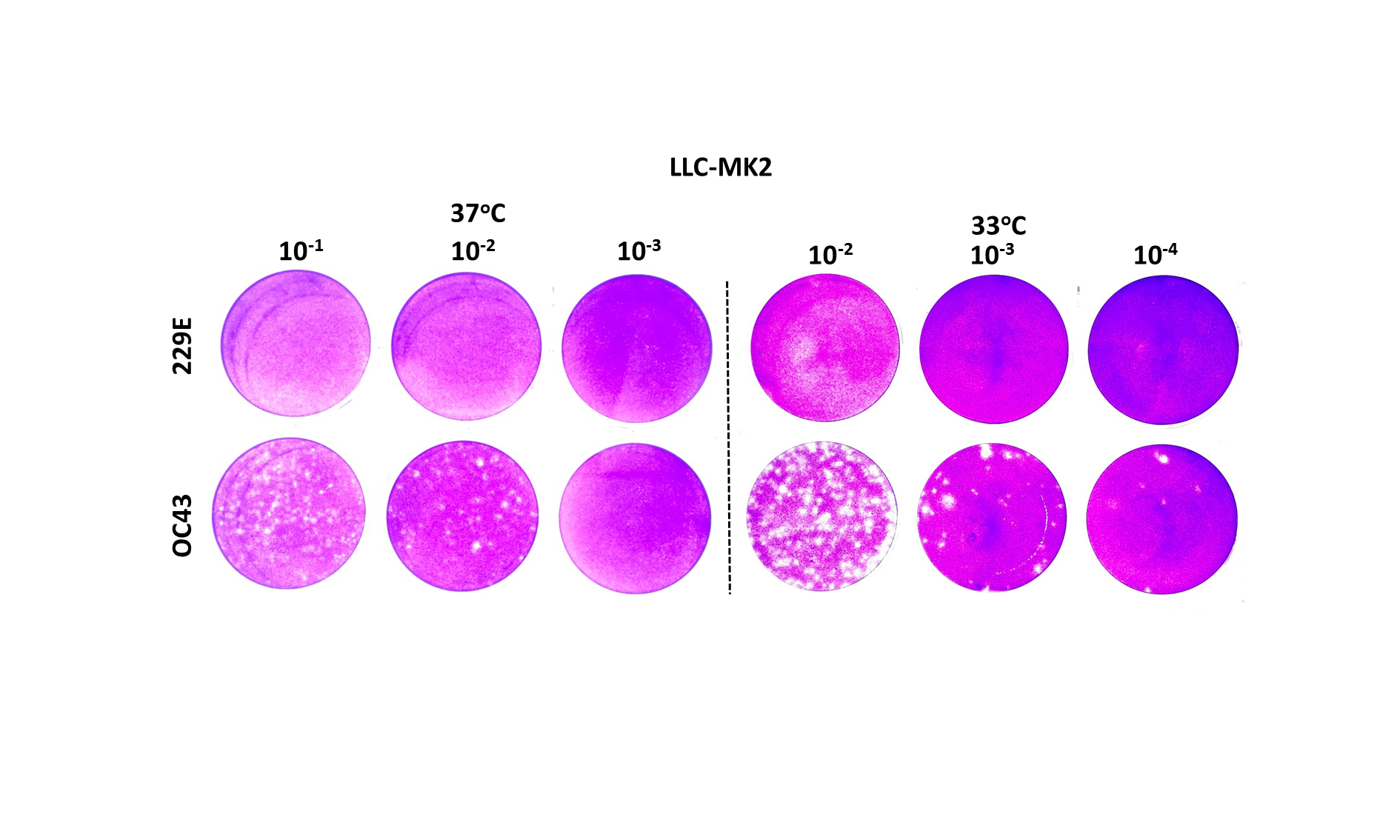

Supplement: Supplemental Information 2 — The plaque assays were incubated at (A) 37C and (B) 33C for five days with a final concentration of 0.3% of agarose and 2.5% of FBS. [file peerj-08-10639-s002.png]

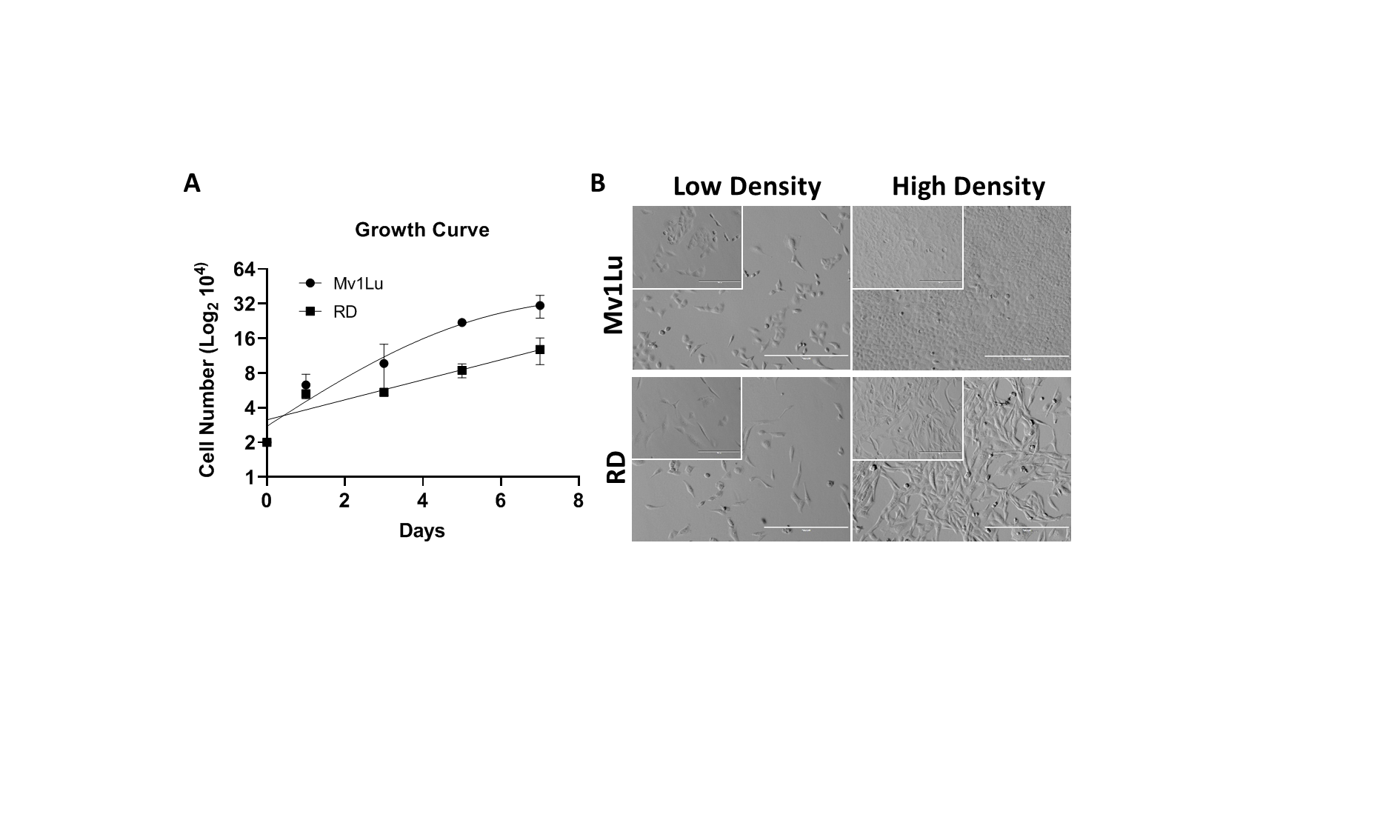

Supplement: Supplemental Information 3 — (A) Growth curve and (B) cell morphology at a low and high density of Mv1Lu and RD cells. Both cells were seeded with 2x10^4^ cells/well and count cell numbers every two days. The data were presented as mean ± SEM and the cell number of y-axis was plotted in log2 scale. Insert images: 200x; background image: 100x. [file peerj-08-10639-s003.png]

Mv1Lu cells can be used in a 12 well plate for titrating OC43 viral titer.

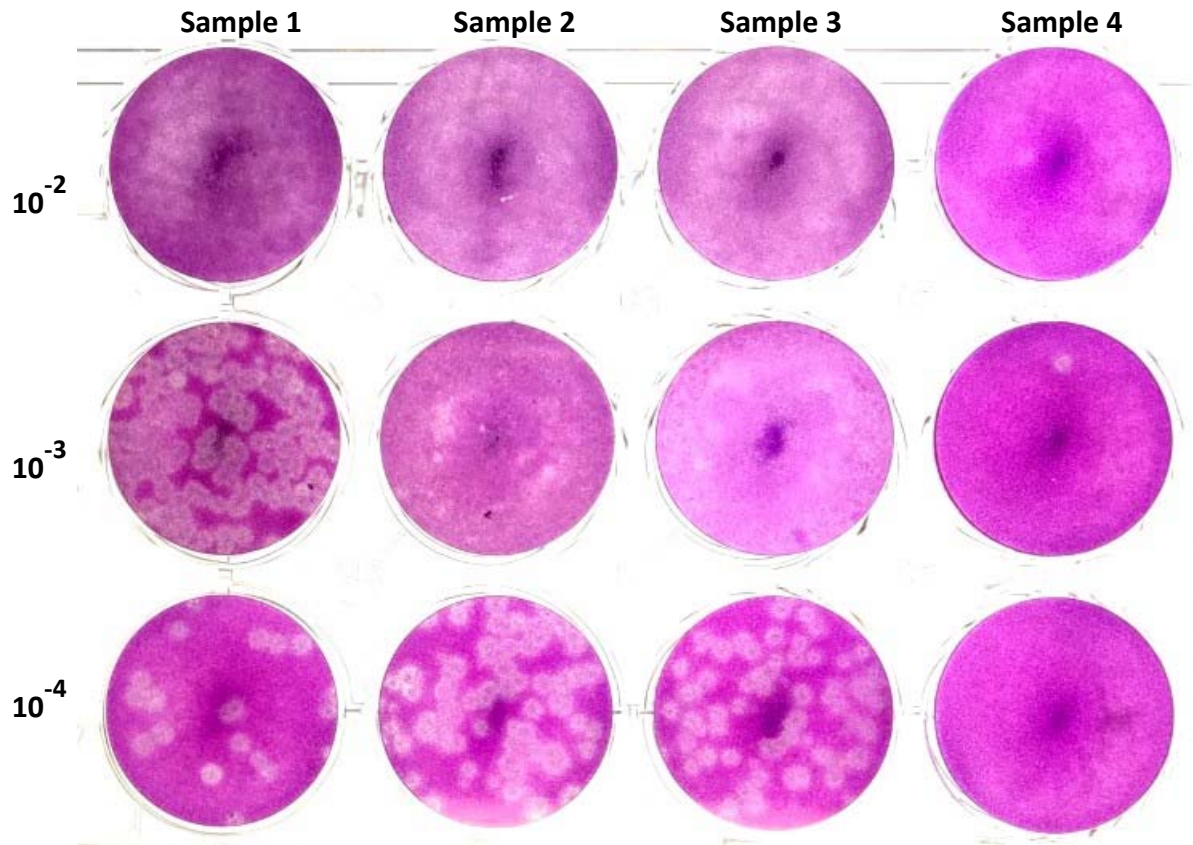

Supplement: Supplemental Information 4 — Mv1Lu were used in a 12-well plate for titrating 3 different OC43 containing samples with various titers. This image different from figures in the main manuscript demonstrates the Mv1Lu cells can be used in a 12 well plate, not 6 well plate, saving reagents and cost. [file peerj-08-10639-s004.pdf]
