# Supplementary material for: Improved plaque assay for human coronaviruses 229E and OC43
Source: PeerJ. 2020 Dec 21;8:e10639. doi: 10.7717/peerj.10639 (PMC7759117; doi:10.7717/peerj.10639)
Supplement: Supplemental Information 5 — Several cell lines were utilized to monitor the cytopathic effects following hCoV infections. CPE is the preliminary indication for a successful plaque assay. [file peerj-08-10639-s005.pdf]

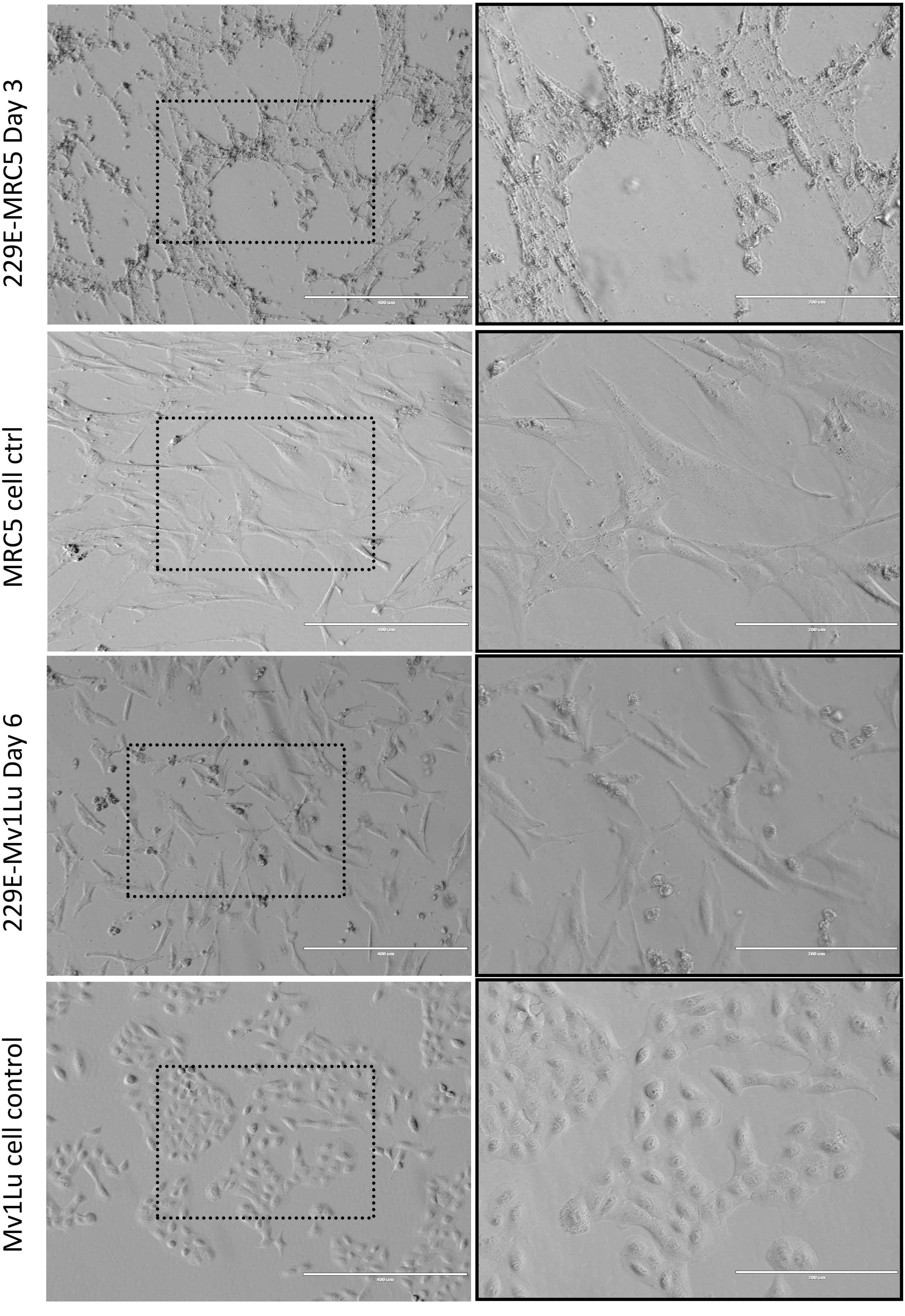
229E-MRC5 Day 3
MRC5 cell ctrl
229E-Mv1Lu Day 6
Mv1Lu cell control

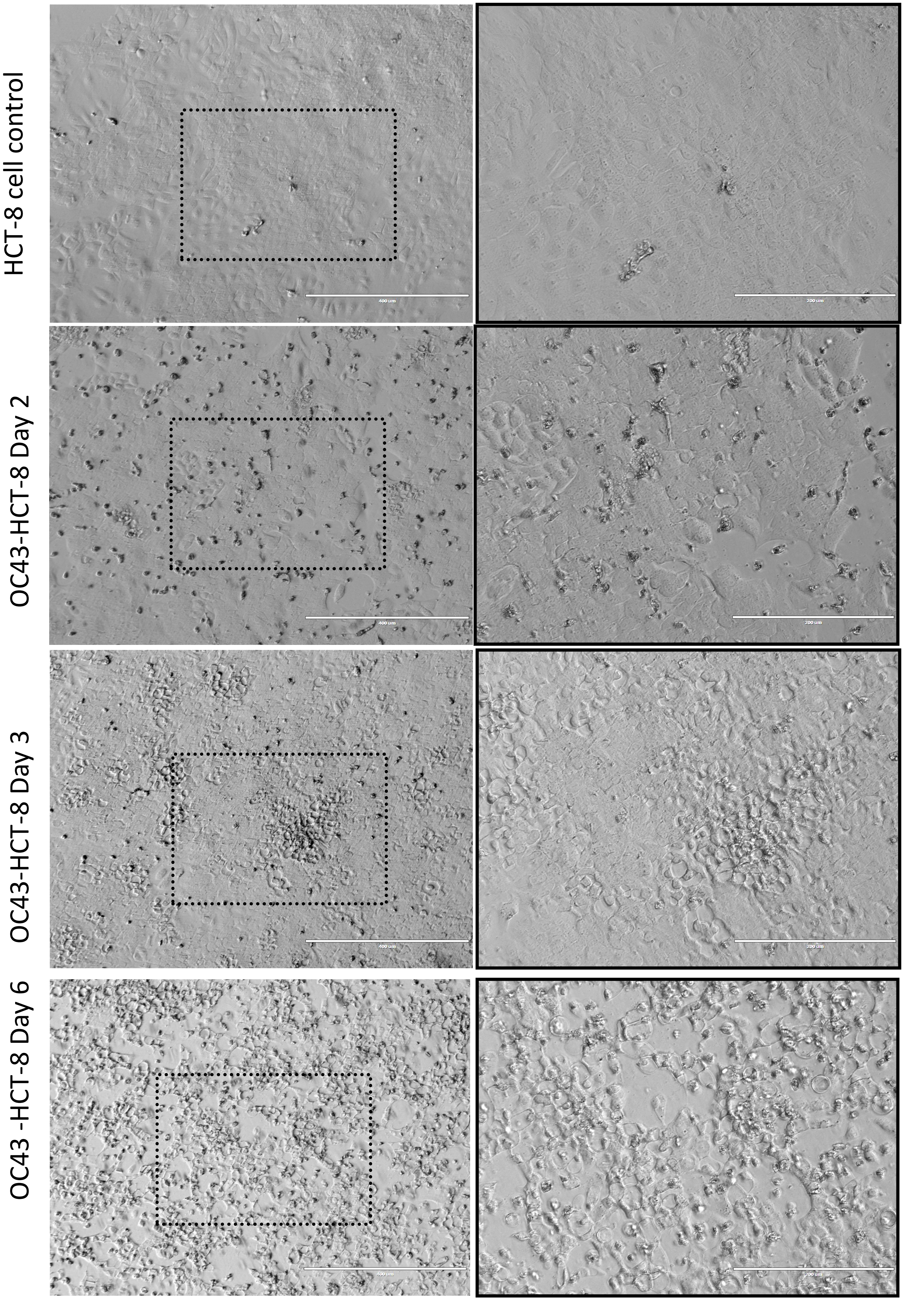
HCT-8 cell control
OC43-HCT-8 Day 2
OC43-HCT-8 Day 3
OC43 -HCT-8 Day 6

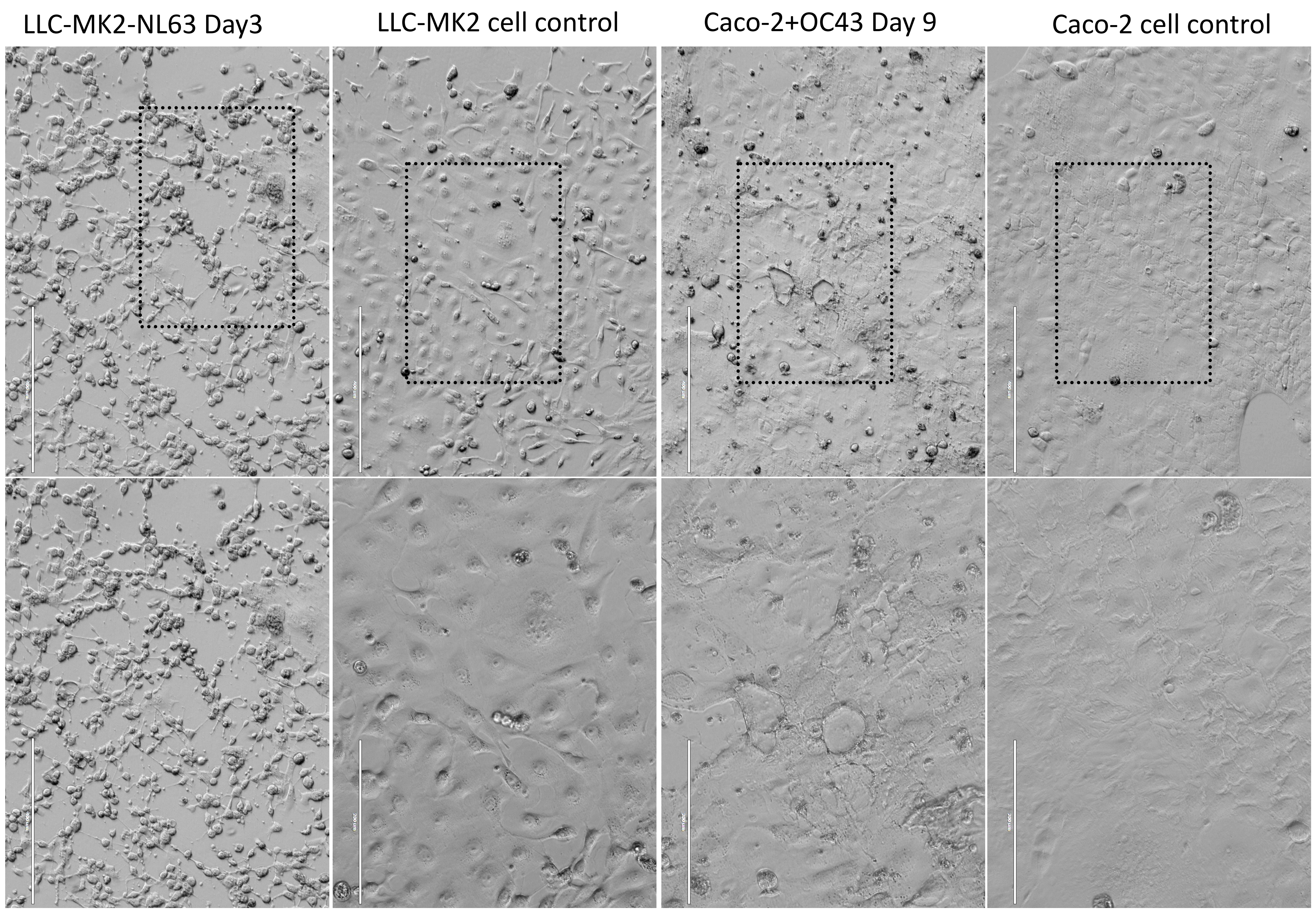
LLC-MK2-NL63 Day3
LLC-MK2 cell control
Caco-2+OC43 Day 9
Caco-2 cell control
